# Composition-based statistics and translated nucleotide searches: Improving the TBLASTN module of BLAST

**DOI:** 10.1186/1741-7007-4-41

**Published:** 2006-12-07

**Authors:** E Michael Gertz, Yi-Kuo Yu, Richa Agarwala, Alejandro A Schäffer, Stephen F Altschul

**Affiliations:** 1 National Center for Biotechnology Information, National Institutes of Health, Department of Health and Human Services, Bethesda, MD, USA

## Abstract

**Background:**

TBLASTN is a mode of operation for BLAST that aligns protein sequences to a nucleotide database translated in all six frames. We present the first description of the modern implementation of TBLASTN, focusing on new techniques that were used to implement composition-based statistics for translated nucleotide searches. Composition-based statistics use the composition of the sequences being aligned to generate more accurate E-values, which allows for a more accurate distinction between true and false matches. Until recently, composition-based statistics were available only for protein-protein searches. They are now available as a command line option for recent versions of TBLASTN and as an option for TBLASTN on the NCBI BLAST web server.

**Results:**

We evaluate the statistical and retrieval accuracy of the E-values reported by a baseline version of TBLASTN and by two variants that use different types of composition-based statistics. To test the statistical accuracy of TBLASTN, we ran 1000 searches using scrambled proteins from the mouse genome and a database of human chromosomes. To test retrieval accuracy, we modernize and adapt to translated searches a test set previously used to evaluate the retrieval accuracy of protein-protein searches. We show that composition-based statistics greatly improve the statistical accuracy of TBLASTN, at a small cost to the retrieval accuracy.

**Conclusion:**

TBLASTN is widely used, as it is common to wish to compare proteins to chromosomes or to libraries of mRNAs. Composition-based statistics improve the statistical accuracy, and therefore the reliability, of TBLASTN results. The algorithms used by TBLASTN are not widely known, and some of the most important are reported here. The data used to test TBLASTN are available for download and may be useful in other studies of translated search algorithms.

## Background

BLAST [1,2] is a popular and effective tool for finding significant alignments between a biological query sequence and a database of subject sequences. BLAST has several modes of operation, one of which aligns an amino acid query sequence to a database of nucleotide sequences, where the nucleotide sequences are often either fragments of a genome or cDNAs representing expressed genes. This mode of operation is known by the name TBLASTN. TBLASTN operates by translating database nucleotide sequences to hypothetical amino acid sequences in all six reading frames and then aligning the hypothetical amino acid sequences to the query.

TBLASTN is widely used as associating proteins with chromosomes or with mRNAs is useful in many biological studies. Despite this popularity, a performance evaluation of TBLASTN has never been published. BLASTX, a related variant of BLAST that aligns a DNA sequence to a protein database, was described in Gish and States[3]. The description of BLASTX in[3], written in 1993, is out-of-date; the paper predates the implementation of gapped alignments in BLAST. We present, in this paper, the first description of the modern implementation of TBLASTN.

An issue of particular concern is the accuracy of the statistics reported by TBLASTN. BLAST prints statistics, most notably an expect value (E-value), to help users evaluate the significance of alignments. These statistics are often used as thresholds to distinguish alignments likely to be due to a biological relationship from alignments that occur simply by chance. It is known, however, that when unrelated amino acid sequences with unusual composition are aligned, alignments with high score occur with implausibly high frequency. In the context of BLASTP, a program that aligns a protein query to a protein database, Schäffer *et al*. [4], Yu *et al*. [5], and Altschul *et al*.[6] describe how to generate amino acid scoring matrices based on the composition of the amino acid sequences being compared. Yu *et al*.[7] show that these compositionally-adjusted matrices yield more accurate statistics for comparing the significance of protein to protein alignments than do the standard matrices.

For TBLASTN, the problem of sequences with biased composition is even worse than for BLASTP. Not only can the hypothetical amino acid sequence contain translations of coding regions for biased proteins, it can also contain regions of compositional bias that are translations of noncoding regions or out-of-frame translations of coding regions. Applying composition-based statistics when one of the sequences is a hypothetical amino acid sequence, however, presents a challenge not present when both sequences are proteins – it is not obvious which parts of the hypothetical sequence to use when computing the composition. For this reason, until recently composition-based statistics have been available only for BLASTP. In this paper, we present methods for adapting composition-based statistics to TBLASTN. We show that these methods improve the statistical accuracy of TBLASTN without unduly affecting retrieval accuracy.

## Results

We have developed techniques and implemented software for applying composition adjustment that takes into account the differences between performing a search against a protein database and performing a search against a translated nucleotide database. We provide a high-level overview of these techniques and their motivation here, but defer a detailed description of the algorithms to the Methods section. We tested the effectiveness of our implementation of composition-based statistics for TBLASTN in two ways. First, we compared the statistical accuracy of several variants of TBLASTN, some of which adjust the scoring system to reflect the composition of the sequences, others of which do not. Second, we performed a ROC (receiver operating characteristic) analysis[8] of the different variants of TBLASTN to test the retrieval accuracy of the variants.

### Compositional adjustment and TBLASTN

There are three critical differences between using a translated nucleotide database and using a database of known proteins or protein fragments. First, each sequence stored in a nucleotide database may contain more than one coding region in the same or different translation frames. Second, the majority of the hypothetical amino acid sequence data generated by translating a nucleotide sequence does not correspond to any protein at all, due to the fact that the location of open reading frames (ORFs) in the nucleotide database is not provided to TBLASTN. One is therefore often either translating a noncoding region or translating a coding region in the wrong frame. Third, the split of a genome into distinct database sequences is performed at sometimes arbitrarily chosen locations, for example when bacterial artificial chromosomes (BACs) are used to obtain the sequence data. Thus, while for BLASTP we use each database sequence in its entirety when computing composition, it would be counterproductive to do so for TBLASTN.

For TBLASTN, we consider "windows" of hypothetical amino-acid data when applying composition based statistics. Each window contains part of a hypothetical coding region for a protein, specifying a substring of nucleotide data and a translation frame. BLAST identifies windows that contain likely coding regions and then uses these windows to compute the composition of the hypothetical proteins. By focusing on smaller regions of the database and frames most likely to contain true amino acids, we capture enough information about the composition of the hypothetical protein to accurately assess the significance of the alignment.

### Variants tested

We tested the statistical and retrieval accuracy of three variants of TBLASTN. All three variants use exactly the same heuristics, as described in the Methods section, to produce a set of windows likely to contain a significant alignment. Then, within each window, the hypothetical subject amino acid sequence is filtered using the SEG[9] algorithm, one of three forms of score adjustment is performed, and alignments are recomputed.

Where the three variants differ is in what type of compositional adjustment is applied. The first version, which we denote here by B-TBLASTN, provides baseline behavior; it ignores the composition of the sequences and merely scales the BLOSUM62[10] matrix to have five more bits of accuracy before rounding. This is performed so that the scores from all three variants tested have comparable scale. The second version, which we denote S-TBLASTN, performs compositional scaling as described in Schäffer *et al*.[4]. The third version, which we denote C-TBLASTN, performs compositional matrix adjustment[5] conditionally as described in Altschul *et al*.[6].

### Tests of statistical accuracy

To evaluate the effect of composition-based statistics on statistical accuracy, we performed a series of tests using randomly permuted sequences. One thousand protein sequences were randomly selected without replacement from the mouse (*Mus musculus*) genome for use as queries. The sequences were permuted using their GenBank identification number as a seed to a random number generator; the permuted sequences are provided as Additional file 1. A permuted sequence necessarily has the same composition as the original sequence, so the query set has the same range of compositions as a sample of true proteins.

The P-value of the highest scoring alignment between each query and the human (*Homo sapiens*) nuclear genome was computed. P-values are related to the E-values reported by BLAST, through the formula

*P *= 1 - *e*^*-E*^

**Figure 1:**
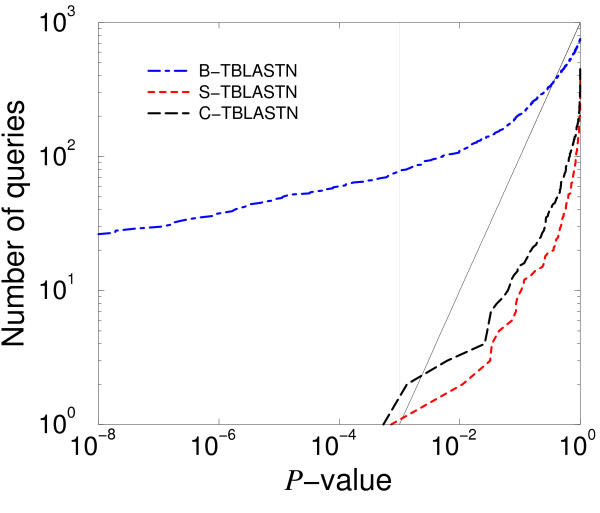
**Statistical accuracy of three variants of TBLASTN**. One thousand queries were randomly selected from mouse proteins, permuted, and aligned to human nuclear DNA. For each variant, we plot against *x *the number of queries with P-value less than or equal to *x*. The solid line is the theoretically ideal distribution of these values.

A P-value represents the probability that an alignment of equal or greater quality will be found when the query and database sequences are unrelated. Figure 1 is a log-log plot of the distribution of P-values for each of the three variants of TBLASTN. The x-axis of the figure is a P-value, and the y-axis is the number of queries whose best match had P-value less than or equal to *x*. For a distribution that matched theory perfectly, the plot would be the straight line that is shown on the figure. For this query set, B-TBLASTN finds many more alignments that have low P-value than theory would predict. For S-TBLASTN and C-TBLASTN, the situation is much better. The plots for both these variants lie mostly to the right of the ideal line, indicating that the statistics are somewhat conservative, but otherwise track the ideal line well.

### ROC analysis of retrieval accuracy

Figure 1 demonstrates the accuracy of P-values when composition-based statistics are used. However, it is known[4,7] that the use of composition-based statistics can have a small, adverse effect on the retrieval accuracy of database searches. This counterintuitive result is thought to be due to the fact that similar compositional bias is itself evidence of biological relatedness[7].

To compare the retrieval accuracy of the three methods, we performed a series of tests using a yeast (*Saccharomyces cerevisiae*) genome. We generated ROC curves and scores by taking a test set, first developed for the study in [11] and later modified and used in [4], updating it to a newer version of the yeast genome, and adapting it for use in TBLASTN. This test set contains 102 protein domains to be used as queries against the yeast nuclear genome. Lists of true matches for each domain against a database of yeast proteins are used to distinguish true from false positive alignments. The lists of true positive matches are human curated. Because the locations of coding regions in yeast DNA are well annotated, we were able to convert the list of true positive protein matches to lists of true positive locations on yeast chromosomes, as described in the Methods section.

**Figure 2:**
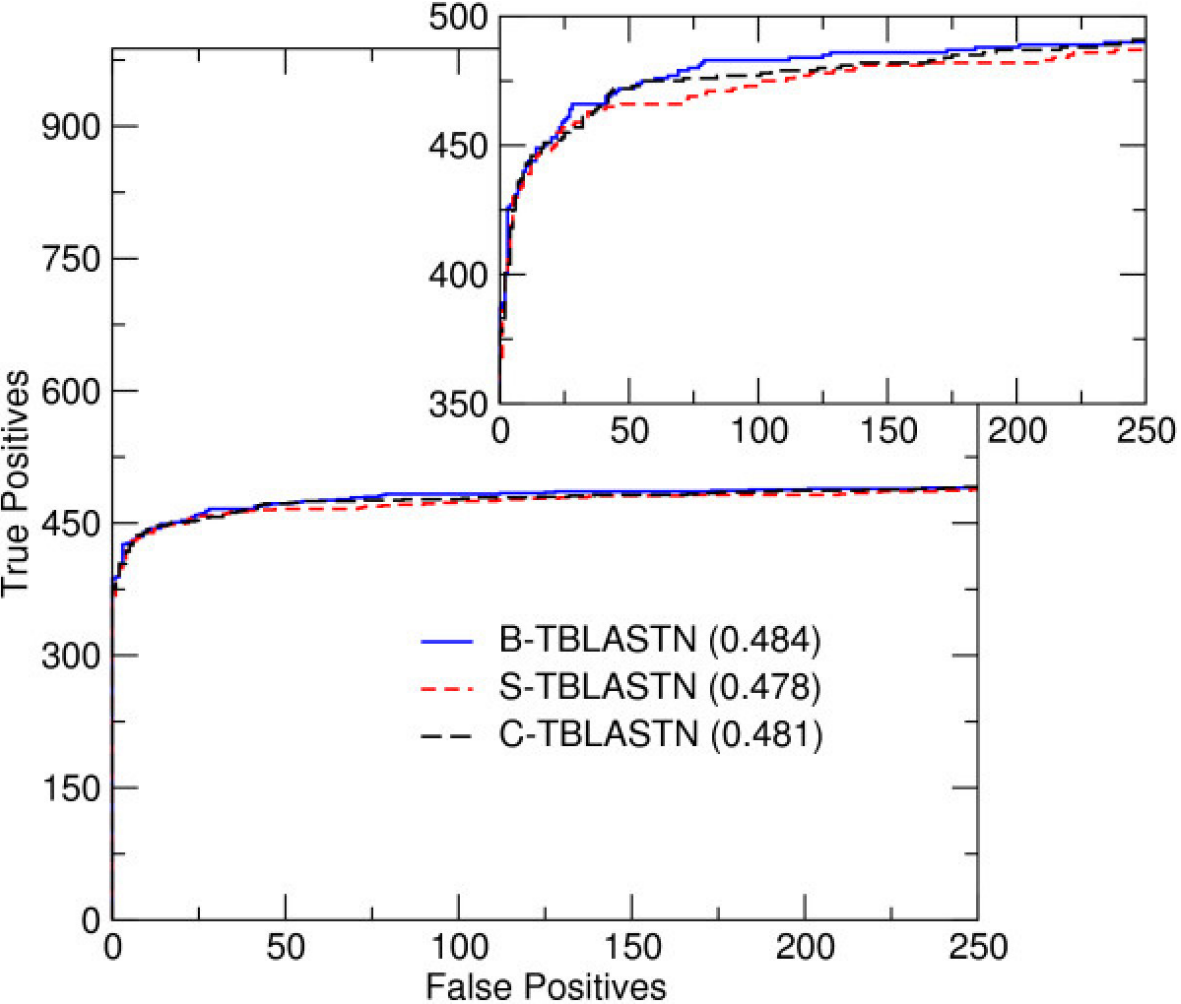
**A portion of the ROC curves for three variants of TBLASTN**. The ROC curves were generated by analyzing the results of aligning 102 queries against the yeast genome. The ROC-250 score for each version of TBLASTN is included in the legend in parentheses after the name of the version. True positives are plotted against false positives, on a linear scale. The total number of true positives possible in this test set was 988. Inset: part of the same ROC curves, plotted on a different scale to show the separation between curves.

**Figure 3:**
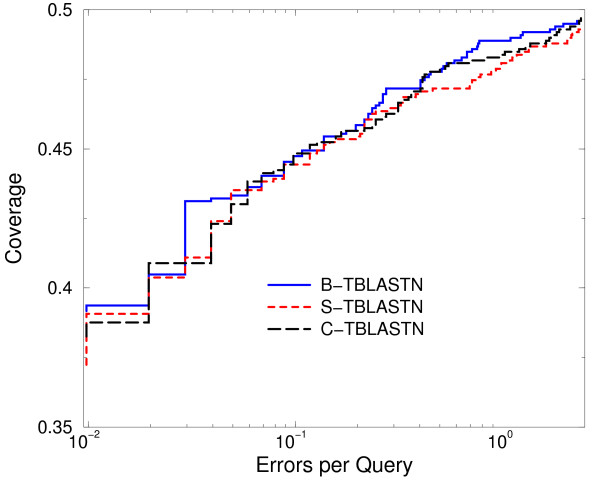
**A semi-log plot of a portion of the ROC curves for three variants of TBLASTN**. The same data as Figure2 in a semi-log plot, using the scales of coverage and errors per query.

Figure 2 shows the ROC curve for each of the three variants tested. Figure 3 shows the same data in a semi-log plot, using the scales of coverage and errors per query. The higher a curve lies in the plot, the better the retrieval accuracy of the method. The B-TBLASTN curve is best, but the S-TBLASTN and C-TBLASTN curves are not excessively low. The differences seen between these curves are comparable to the difference one would expect to see, based on similar tests of protein-protein searches[7]. Conditional compositional matrix adjustment, the method used by C-TBLASTN, shows better retrieval accuracy than S-TBLASTN. These results are consistent with the trend reported in[6]. To quantify the difference between the three methods, Table 1 shows the ROC scores for the three methods, computed at several thresholds of false positive matches. Table 1 shows the same trend as Figures 2 and 3.

**Table 1:** ROC scores for three variants of TBLASTN. ROC scores for three variants of TBLASTN, at several thresholds of false positive matches. These scores were generated by analyzing the results of aligning 102 queries against the yeast genome.

Program	50	150	250
B-TBLASTN	0.458 ± 0.004	0.478 ± 0.002	0.484 ± 0.001
S-TBLASTN	0.454 ± 0.004	0.471 ± 0.002	0.478 ± 0.001
C-TBLASTN	0.455 ± 0.004	0.474 ± 0.002	0.481 ± 0.001

## Discussion

We have integrated composition-based statistics into recent versions of TBLASTN. Another contribution of our work is the development of a test set for translated searches (this test set is provided as Additional file 2. Our tests show that use of composition-based statistics with TBLASTN improves the statistical accuracy of the algorithm. It does, however, have a small negative effect on the retrieval accuracy.

Retrieval accuracy measures the ability of a method to find true positive matches and rank them before false positives matches. Statistical accuracy provides a means to distinguish the two groups by choosing a reasonable E-value cutoff. Because of the tendency, shown in Figure 1, for TBLASTN to overstate the significance of alignments with biased composition, the use of compositional adjustment improves the reliability of the method when it is used with an E-value cutoff. In many applications, it is of particular importance to exclude false positive matches. For example, in PSI-BLAST, the inclusion of false positive matches in the results may corrupt the generated profile[4]. Therefore, we are willing to tolerate a small decline in retrieval accuracy for the sake of statistical accuracy.

Without composition-based statistics, the only way to use an E-value cutoff to exclude false positive matches with biased composition is to set the cutoff to a small number. However, such a cutoff is too stringent for the majority of matches that do not have strong bias. With composition-based statistics, one can more confidently take E-values at face value. The statistical interpretation of E-values then provides guidance on how to choose E-value cutoffs. For example, with perfect statistics, if one were performing 1000 tests, one would expect to find one false positive if the E-value cutoff were set to 10^-3^. Figure 1 shows that TBLASTN with composition based statistics produces E-values that are somewhat conservative. Therefore, the E-value cutoff may exclude some marginal true positives. In many applications, it is worth excluding a few marginal true positives to exclude the large number of false positives shown in Figure 1.

TBLASTN uses the BLAST heuristics to perform a database search and so is capable of searching large nucleotide databases relatively quickly. By focusing on applying fast heuristics in six reading frames, however, TBLASTN does not directly address some phenomena commonly found in nucleotide databases. Databases of expressed sequence tags (ESTs)[12,13] contain "single-pass" cDNA sequences that frequently contain frameshift errors. Each distinct alignment reported by TBLASTN uses only one of the possible reading frames of the DNA sequence; TBLASTN does not explicitly consider the possibility that an alignment could be extended in another frame. The local algorithms used by BLAST also do not search for splice sites or explicitly try to distinguish introns from exons.

TBLASTN includes heuristics that indirectly address the issues of frame shifts and introns, without compromising speed. TBLASTN uses a greedy algorithm to link nearby distinct alignments and computes an E-value for these linked sets[14]. Alignments are eligible to be linked if they are in any of the three reading frames on the same strand of DNA. Therefore, some cases of frameshifts can be found. Moreover, an option may be set so that the algorithm links alignments separated by relatively long stretches of unaligned DNA. If this option is set, TBLASTN is sometimes able to link exons of a gene together. However, frameshifts and exons are not labeled as such by TBLASTN, and the only evidence that the sets are correctly linked is that they are in a consistent order and yield a lower E-value than each alignment would if taken on its own.

By contrast, there are other software packages that do make a special effort to handle frameshifts and possibly introns. To our knowledge, the earliest translated search algorithm is described by Peltola *et al*.[15], which explicitly treats frameshifts and introns as part of a dynamic program. Several other methods based on dynamic programming have been proposed [16-23]. These methods generally make frameshifts or introns, or both, part of the dynamic programming formulation.

The FASTA package[24] has for some time implemented a version that aligns a protein to a nucleotide sequence translated in either three or six reading frames. The methods described in[21] and[22], which are able to align through frameshifts, have been incorporated into the FASTA package. The relevant dynamic programs are approximately solved using the FASTA heuristics.

References [15,17-20,23] do not discuss the statistics reported by the algorithm, and we are not aware of any reference to what statistics they propose to report. None indicate that they wish to take the composition of the sequences into account when evaluating significance. The method described in[16] is based on Bayesian probabilities, but does not take amino acid composition into account. References describing translated searches in FASTA[21,22,24] do not describe the statistics reported, but the use of statistics in FASTA is well documented[25]. FASTA does not use any notion similar to composition-based statistics, but rather uses *a posteriori *statistics based on the actual scores of alignments found for a particular query. Therefore, the composition of the query indirectly affects the statistics reported, as it affects the observed distribution of scores. We prefer an *a priori *approach explicitly using the composition of the sequences being compared, particularly because this approach can take the composition of regions of the subject sequence into account.

Hein[26] and Gelfand *et al*.[27] describe algorithms that handle introns and frameshifts by linking multiple distinct alignments. TBLASTN is similar in that it also uses linking, though the algorithms used to perform the linking are quite different. Hein describes an algorithm similar to BLASTX[3] that joins ungapped alignments. There is no explicit treatment of frameshifts. Gelfand *et al*. use a set of heuristics, based in part on the presence of splice sites, to create a collection of ranges in the nucleotide sequence that they consider most likely to be coding exons of a gene. They translate each block in one reading frame and perform a gapped alignment to a query protein. Then, they link distinct blocks together to predict a complete gene. Neither[26] or[27] discusses statistics or make any mention of using composition when evaluating the significance of alignments.

Another approach to aligning a protein to a nucleotide sequence is to use Hidden Markov Models (HMMs). This is the approach taken by Birney *et al*.[28,29] in the package GeneWise and Halperin *et al*.[30] in the package FramePlus. Both packages use HMMs to model nucleotide sequences, and explicitly include states and transitions that model frameshifts and intron-exon structure. Durbin *et al*.[31] explain how to use the transition probabilities in a profile HMM to arrive at a P-value for an alignment.

There has also been interest in aligning nucleotide sequences by translating both sequences in all six reading frames and aligning the resulting amino acid sequences. BLAST, BLAT[32] and MUMmer[33,34] all have modes of operation that align nucleotide sequences in this fashion. BLAT also has modes of operation that align protein sequences to translated nucleotide sequences, though these modes are not described in[32]. BLAT is designed to find high percent identity matches quickly, whereas BLASTX and TBLASTN are designed to perform a thorough search for homologs. Because of its different purpose, BLAT finds many fewer alignments than does TBLASTN. In default mode on the yeast test, BLAT finds only 54 true positives in total, whereas B-TBLASTN finds 386 before the first false positive. To our knowledge, neither BLAT nor MUMmer takes any special action to handle introns or frameshifts when operating in translated mode. MUMmer does not report P-values or E-values for alignments, nor adjusts scores to account for composition. BLAT does not report E-values in its default output format, but provides E-values in alternate output formats that roughly mimic output formats provided by BLAST.

The lack of explicit treatment of frameshifts and introns must be considered a disadvantage of TBLASTN. Further research into how TBLASTN can be modified to handle these issues is indicated. We emphasize, however, that the improvement in statistical accuracy reported in this paper is a step toward that goal. Accurate statistics improve reliability in any application in which an E-value cutoff is used. This reduces the number of possibilities that must be considered when evaluating intron-exon structure or testing for the presence of frameshifts.

Other ways to improve the quality of TBLASTN results are under investigation, including the algorithm and data structure used to find the initial short matches, sometimes called "seeds". For example, Brejová *et al*.[35] proposed various discontiguous seed patterns with "don't care" positions and did a large-scale test to suggest that these would improve performance of BLASTP (and hence, likely also TBLASTN).

## Conclusion

We present here the modern implementation of TBLASTN and describe how composition-based statistics, previously available only for protein searches against a protein database, has been made available for TBLASTN. We show that composition-based statistics improve the statistical accuracy, and therefore the reliability, of TBLASTN results, with only a small loss in retrieval accuracy.

TBLASTN is widely used, as it is common to wish to compare proteins to chromosomes or to libraries of mRNAs. The primary advantages of TBLASTN are that it is well-supported, widely and freely available, under active development, and integrated with other NCBI tools. TBLASTN may be used from NCBI's web page[36], where it is linked with NCBI databases and other resources. BLAST is supported by the NCBI Help Desk and a dedicated group of software engineers and information technology specialists. NCBI provides on-line tutorials and face-to-face workshops on the use of BLAST and other NCBI resources; see[37]. Details of the algorithms used by TBLASTN, however, have not previously been published and are not widely known.

The test sets and methodology discussed in this paper may be useful in other studies of translated nucleotide search algorithms. We have made the data used to perform the ROC analysis of TBLASTN available as Additional file 2.

The advances described in this paper have been integrated into the NCBI C and C++ software distributions; the computational modules involved are mirrored between the two distributions. Options for applying composition-based statistics with TBLASTN are available on NCBI's BLAST web page[36]. A command-line executable, blastall, that has TBLASTN as one of its modes of operation, is available for download from the same URL. Source code and precompiled executables for some platforms are provided. We describe the options required to make blastall run TBLASTN with composition-based statistics in the Methods section.

## Methods

In this section, we outline the algorithm used to compute the composition of database sequences and to apply composition-based statistics in TBLASTN. Then we further describe the tests reported in this paper: the executables used, the tests sets, and details about the methods.

### Compositional adjustment in TBLASTN

The BLAST heuristics[2] use a general scoring system, such as the PAM[38,39] or BLOSUM[10] series of matrices, to discover database sequences likely to align to the query and likely starting points for alignments. In BLAST, an alignment is known as a high-scoring pair, or HSP[40]. A list of HSPs for each significant query-subject pair is created using a multi-stage algorithm. At each stage, HSPs may be culled from the current list for a number of reasons, including having insufficiently high score, being contained in a higher-scoring HSP, or sharing an endpoint with a higher-scoring HSP. As a result, while each successive stage of the BLAST algorithm requires significantly more computation for each HSP, fewer HSPs need be considered.

Compositional adjustment, whether used by TBLASTN or other modes of operation, is applied only in the final stage of a BLAST search. In this fashion, modes that use compositional adjustment apply the fast heuristics of BLAST to locate regions likely to contain, and starting points likely to lead to, high-scoring alignments. They apply compositional adjustment only before the most sensitive and most computationally expensive alignment algorithm, the computation of a gapped alignment that includes information specifying the locations of gaps, information known as the "traceback". The list of HSPs produced by this final gapped alignment, after being filtered for insufficiently significant or redundant HSPs, is the list presented to the user.

### Steps for applying compositional adjustment

At a high level, the steps of composition adjustment, applied individually to each query-subject pair, are as follows: (1) compute windows of interest using the list of HSPs from preliminary stages of the BLAST algorithm; (2) obtain translated subject data for the windows and filter it to remove uninteresting subsequences; (3) compute the composition of the subject region for each HSP to be realigned; (4) compute a scoring matrix for each HSP to be realigned, based on the composition of the subject region of that HSP and on the composition of the query; (5) perform a gapped alignment with traceback to recompute the list of HSPs, using the new scoring matrices. In practice, these high-level steps are interleaved to reduce memory requirements.

### Computing windows of interest

For each match between the query and a subject sequence, the compositional adjustment algorithm is given a separate list of HSPs. Each HSP specifies, along with other information, a range in the subject sequence that has been aligned to the query. These ranges are used as follows to compute a list of windows. First, a preliminary list of windows for the subject sequence is created. This list contains one window for each HSP, the window that surrounds the subject range of the HSP, including 600 bases to the left and right of the subject range if that much sequence data is available. Then a final list of windows is created by joining windows in the same translation frame if they touch or overlap. For each window, a list of HSPs corresponding to the window is maintained.

### Obtaining and filtering subject data

The nucleotide subject data within a window is obtained and translated using that window's translation frame. The SEG[9] algorithm with window size 10, low-cutoff 1.8, and high cutoff 2.1 is used to mask low-complexity regions in the subject window. The parameters were chosen as a result of the study[4]. A low-complexity region is typically dominated by a few distinct residues often, but not always, in a repetitive pattern. Typical examples are polyglycine or polyproline monomers. Alignment scores that include the scores of low-complexity regions tend to overstate the significance of the alignments and lead to many false positive matches.

The effect of applying the SEG algorithm to an amino acid sequence is to replace each residue in a low-complexity region with the X character: a character that is assigned a small negative score when aligned to any character, including itself. The subject data are filtered before compositionally adjusted scoring matrices are computed, and occurrences of the X character are ignored when computing the composition of a sequence. Unlike the composition-adjustment code, the preliminary stages of the BLAST search do not filter the subject data.

SEG filtering may also be applied to the query sequence. SEG filtering of the query is a command-line option for both BLASTP and TBLASTN. The programs differ in that SEG filtering of the query is off by default in BLASTP but on by default in TBLASTN. We did not filter the query in any results reported in this paper. The SEG parameters used to filter the subject sequence apply a higher threshold for declaring a region to be low-complexity than do the default parameters used to filter the query. The reason that the query sequence is more stringently filtered is that the query sequence is used at every stage of the BLAST algorithm. SEG filtering of the subject only occurs at the final stages of a BLAST search, and under-filtering the data within a subject window will effect only a single comparison.

### Computing the composition of the subject

For TBLASTN, the sequence data and the subject ranges of the HSPs within a window are used to determine a range likely to contain correctly translated amino acid data. The window is searched strictly to the left of the subject range of the HSP to find the rightmost occurrence of a stop codon. If one is found, then the location 20 characters to the right of the stop codon is the left boundary of the composition range, with the restriction that the entire subject range of the HSP be included. If no stop codon is found, then the left endpoint is the left endpoint of the window. The symmetric rule is applied to the right.

The intent is not necessarily to locate the stop codon that terminates the protein, but rather to use the presence of a stop codon to indicate that the hypothetically translated codon lies in a noncoding region. Indeed, the noncoding region may be an intron rather than the true end of the amino acid sequence. Because we are not attempting to find a terminating stop codon, we propose a symmetric rule to determine the sequence range to use for composition adjustment even though biological translation is asymmetric.

In a random DNA sequence with 50% GC content, one would expect to find a stop codon in a hypothetically translated amino acid sequence on average once every 21 characters. Therefore, we institute a 20 character margin between the stop codon and the range to use for composition adjustment, with the restriction that the entire subject range of the HSP be included.

Given a particular region, TBLASTN considers only the 20 standard amino acids when computing composition; the X character, the stop character, and all other nonstandard characters are completely ignored. When the length of the sequence is used in the compositional adjustment algorithms, the value used does not count occurrences of ignored characters.

### Computing compositionally-adjusted scoring matrices

Schäffer *et al*.[4] and Yu *et al*.[5] show how to adjust substitution scores for the 20 standard amino acids. For the standard amino acids, we apply those techniques. These papers do not, however, discuss the treatment of rarely occurring amino acids, two-letter ambiguity characters, the X character, or the stop character. We discuss the treatment of the X and stop characters in this section, because they occur commonly in TBLASTN searches. We discuss the treatment of the other characters in Additional file 3.

The stop character occurs frequently in translated sequences and occasionally within significant alignments. An occurrence of the stop character usually indicates that one is translating a noncoding region or a coding region in the wrong frame. Of course, a stop character can also simply mark the end of translation. However, stop characters occur within significant alignments for several reasons: the subject sequence may contain a pseudogene; the subject sequence may be mitochondrial DNA, in which certain codons that are stop codons in nuclear DNA are translated to true amino acids [41-43]; the subject sequence may contain a stop codon that is converted *in vivo *to a selenocysteine[44,45] or pyrrolysine[46] residue; the subject sequence may represent a gene, such as the *hdc *gene in *D. melanogaster*[47,48], that encodes a protein product by mRNA readthrough; or there may be a sequencing error in the subject sequence.

Appropriate scoring of the stop character is essential to TBLASTN. Any character aligned to a stop character should be given a negative score, but not a negative score of such large magnitude as to disallow valid alignments containing a stop codon. BLAST uniformly assigns letters aligned to a stop codon an integral score that, given the scale being used, is as close as possible to -2 bits.

As just discussed, biologically meaningful and statistically significant TBLASTN alignments may sometimes contain translated stop codons. However, the presence of many stop codons in noncoding regions and out-of-frame coding regions renders it very unlikely that these regions will yield high-scoring alignments by chance. Accordingly, for E-value calculations, TBLASTN assumes the length of a database sequence to be the length of the protein yielded by translation in a single reading frame, even though translation is in fact performed in all six reading frames. That many database DNA sequences are noncoding over much of their lengths may be one explanation for the generally conservative statistics of S-TBLASTN and C-TBLASTN shown in Figure 1.

Because of the application of the SEG algorithm, the X ambiguity character is common, and the treatment of X characters can significantly effect the performance of the algorithm. We score alignments with X as follows. When either compositional matrix scaling or compositional matrix adjustment is used, substitution scores are computed for all standard amino acids. Then, for all variants tested here, the score of aligning a standard amino acid *i *in the query sequence to an X in the subject is computed using the formula:

score(i,X)=round(min⁡[−1,∑j∈Sscore(i,j)×P′j]),     (1)
 MathType@MTEF@5@5@+=feaafiart1ev1aaatCvAUfKttLearuWrP9MDH5MBPbIqV92AaeXatLxBI9gBamXvP5wqSXMqHnxAJn0BKvguHDwzZbqegyvzYrwyUfgarqqtubsr4rNCHbGeaGqiA8vkIkVAFgIELiFeLkFeLk=iY=Hhbbf9v8qqaqFr0xc9pk0xbba9q8WqFfeaY=biLkVcLq=JHqVepeea0=as0db9vqpepesP0xe9Fve9Fve9GapdbaqaaeGacaGaaiaabeqaamqadiabaaGcbaGaee4CamNaee4yamMaee4Ba8MaeeOCaiNaeeyzau2aaeWaaeaacqWGPbqAcqGGSaalcqWGybawaiaawIcacaGLPaaacqGH9aqpcqqGYbGCcqqGVbWBcqqG1bqDcqqGUbGBcqqGKbazdaqadaqaaiGbc2gaTjabcMgaPjabc6gaUnaadmaabaGaeyOeI0IaeGymaeJaeiilaWYaaabeaeaacqqGZbWCcqqGJbWycqqGVbWBcqqGYbGCcqqGLbqzdaqadaqaaiabdMgaPjabcYcaSiabdQgaQbGaayjkaiaawMcaaiabgEna0kqbdcfaqzaafaaaleaacqWGQbGAcqGHiiIZcqWGtbWuaeqaniabggHiLdGcdaWgaaWcbaGaemOAaOgabeaaaOGaay5waiaaw2faaaGaayjkaiaawMcaaiabcYcaSiaaxMaacaWLjaWaaeWaaeaacqaIXaqmaiaawIcacaGLPaaaaaa@768B@

where S
 MathType@MTEF@5@5@+=feaafiart1ev1aaatCvAUfKttLearuWrP9MDH5MBPbIqV92AaeXatLxBI9gBamrtHrhAL1wy0L2yHvtyaeHbnfgDOvwBHrxAJfwnaebbnrfifHhDYfgasaacH8akY=wiFfYdH8Gipec8Eeeu0xXdbba9frFj0=OqFfea0dXdd9vqai=hGuQ8kuc9pgc9s8qqaq=dirpe0xb9q8qiLsFr0=vr0=vr0dc8meaabaqaciaacaGaaeqabaWaaeGaeaaakeaaimaacqWFse=uaaa@3845@ is the set of standard amino acids and P′j
 MathType@MTEF@5@5@+=feaafiart1ev1aaatCvAUfKttLearuWrP9MDH5MBPbIqV92AaeXatLxBI9gBaebbnrfifHhDYfgasaacH8akY=wiFfYdH8Gipec8Eeeu0xXdbba9frFj0=OqFfea0dXdd9vqai=hGuQ8kuc9pgc9s8qqaq=dirpe0xb9q8qiLsFr0=vr0=vr0dc8meaabaqaciaacaGaaeqabaqabeGadaaakeaacuWGqbaugaqbamaaBaaaleaacqWGQbGAaeqaaaaa@2F6A@ is the probability of amino acid *j *in the subject sequence. In other words, the score of matching a standard amino acid with X is the expected value over all matches of that amino acid with a standard amino acid, provided that this value is less than -1. For B-TBLASTN and S-TBLASTN, P′j
 MathType@MTEF@5@5@+=feaafiart1ev1aaatCvAUfKttLearuWrP9MDH5MBPbIqV92AaeXatLxBI9gBaebbnrfifHhDYfgasaacH8akY=wiFfYdH8Gipec8Eeeu0xXdbba9frFj0=OqFfea0dXdd9vqai=hGuQ8kuc9pgc9s8qqaq=dirpe0xb9q8qiLsFr0=vr0=vr0dc8meaabaqaciaacaGaaeqabaqabeGadaaakeaacuWGqbaugaqbamaaBaaaleaacqWGQbGAaeqaaaaa@2F6A@ is the actual frequency of the amino acid in the subject region; for C-TBLASTN, the probabilities are computed using pseudocounts, as described in [6]. A formula analogous to Equation(1) is used to compute the score of aligning an X character in the query to a standard amino acid in the subject. The score for aligning X to itself is the smaller of the expected score of aligning any two standard amino acids and -1, rounded to the nearest integer.

### Performing a gapped alignment with traceback

Routines that apply composition-based statistics do not merely rescore alignments, but rather recompute them. Alignments are computed using one of two techniques. By default, the x-drop algorithm [2,49] is applied to a set of starting points specified in the lists of HSPs provided from previous stages of the BLAST algorithm[1,2]. As a result of modifications made during the course of this project, one may alternately specify that the rigorous Smith-Waterman[50] algorithm be applied within each window. If the x-drop algorithm is applied, the composition is computed individually for each HSP that is realigned. If the Smith-Waterman algorithm is used, the composition of a window is taken to be the composition of its highest-scoring HSP. Pooling the composition of the subject regions of several HSPs within a window is problematic because the HSPs do not necessarily belong to the same alignment, or even to the same linked set of alignments. The default in TBLASTN is to use the x-drop algorithm, and we use the x-drop algorithm in the tests presented in this paper.

The following pseudocode shows how alignments corresponding to a single query-subject match are recomputed when the x-drop algorithm is used. In the pseudocode, *q *represents the query data, W
 MathType@MTEF@5@5@+=feaafiart1ev1aaatCvAUfKttLearuWrP9MDH5MBPbIqV92AaeXatLxBI9gBamrtHrhAL1wy0L2yHvtyaeHbnfgDOvwBHrxAJfwnaebbnrfifHhDYfgasaacH8akY=wiFfYdH8Gipec8Eeeu0xXdbba9frFj0=OqFfea0dXdd9vqai=hGuQ8kuc9pgc9s8qqaq=dirpe0xb9q8qiLsFr0=vr0=vr0dc8meaabaqaciaacaGaaeqabaWaaeGaeaaakeaaimaacqWFwe=vaaa@384D@ is a list of windows, D
 MathType@MTEF@5@5@+=feaafiart1ev1aaatCvAUfKttLearuWrP9MDH5MBPbIqV92AaeXatLxBI9gBamrtHrhAL1wy0L2yHvtyaeHbnfgDOvwBHrxAJfwnaebbnrfifHhDYfgasaacH8akY=wiFfYdH8Gipec8Eeeu0xXdbba9frFj0=OqFfea0dXdd9vqai=hGuQ8kuc9pgc9s8qqaq=dirpe0xb9q8qiLsFr0=vr0=vr0dc8meaabaqaciaacaGaaeqabaWaaeGaeaaakeaaimaacqWFdepraaa@3827@ is a source of sequence data, and *params *is a structure containing all parameters needed for gapped alignment. The variable A
 MathType@MTEF@5@5@+=feaafiart1ev1aaatCvAUfKttLearuWrP9MDH5MBPbIqV92AaeXatLxBI9gBamrtHrhAL1wy0L2yHvtyaeHbnfgDOvwBHrxAJfwnaebbnrfifHhDYfgasaacH8akY=wiFfYdH8Gipec8Eeeu0xXdbba9frFj0=OqFfea0dXdd9vqai=hGuQ8kuc9pgc9s8qqaq=dirpe0xb9q8qiLsFr0=vr0=vr0dc8meaabaqaciaacaGaaeqabaWaaeGaeaaakeaaimaacqWFaeFqaaa@3821@ represents the new set of alignments to be returned, and *M *represents a compositionally adjusted scoring matrix. The HSP_IS_CONTAINED and WITH_DISTINCT_ENDS routines will be described below; the action of the remaining routines should be clear from their names.

### Algorithm 1

Redo alignments in a window.

**function **REDO_ONE_WINDOW (*q*, *w*, D
 MathType@MTEF@5@5@+=feaafiart1ev1aaatCvAUfKttLearuWrP9MDH5MBPbIqV92AaeXatLxBI9gBamrtHrhAL1wy0L2yHvtyaeHbnfgDOvwBHrxAJfwnaebbnrfifHhDYfgasaacH8akY=wiFfYdH8Gipec8Eeeu0xXdbba9frFj0=OqFfea0dXdd9vqai=hGuQ8kuc9pgc9s8qqaq=dirpe0xb9q8qiLsFr0=vr0=vr0dc8meaabaqaciaacaGaaeqabaWaaeGaeaaakeaaimaacqWFdepraaa@3827@, *params*, *cutoff_score*)

   A
 MathType@MTEF@5@5@+=feaafiart1ev1aaatCvAUfKttLearuWrP9MDH5MBPbIqV92AaeXatLxBI9gBamrtHrhAL1wy0L2yHvtyaeHbnfgDOvwBHrxAJfwnaebbnrfifHhDYfgasaacH8akY=wiFfYdH8Gipec8Eeeu0xXdbba9frFj0=OqFfea0dXdd9vqai=hGuQ8kuc9pgc9s8qqaq=dirpe0xb9q8qiLsFr0=vr0=vr0dc8meaabaqaciaacaGaaeqabaWaaeGaeaaakeaaimaacqWFaeFqaaa@3821@←∅

   *H*←*windows.hsps*

   SORT_BY_SCORE(*H*)

   *s*←GET_TRANSLATED_SUBJECT (*w*, D
 MathType@MTEF@5@5@+=feaafiart1ev1aaatCvAUfKttLearuWrP9MDH5MBPbIqV92AaeXatLxBI9gBamrtHrhAL1wy0L2yHvtyaeHbnfgDOvwBHrxAJfwnaebbnrfifHhDYfgasaacH8akY=wiFfYdH8Gipec8Eeeu0xXdbba9frFj0=OqFfea0dXdd9vqai=hGuQ8kuc9pgc9s8qqaq=dirpe0xb9q8qiLsFr0=vr0=vr0dc8meaabaqaciaacaGaaeqabaWaaeGaeaaakeaaimaacqWFdepraaa@3827@)

   **for ***i*←0 **to ***length*(*H*)-1 **do**

      *h*←*H[i]*

      **if forall **0≤ *j *<*i ***not **HSP_IS_CONTAINED(*h*, *H *[*j*]) **then**

         *M*←ADJUST_COMPOSITION (*q*, *s*, *h*, *params*)

         *a*←CALC_X_DROP_ALIGNMENT (*q*, *s*, *h*, *M*, *params*)

         **if ***a.score≥cutoff_score ***then**

            A
 MathType@MTEF@5@5@+=feaafiart1ev1aaatCvAUfKttLearuWrP9MDH5MBPbIqV92AaeXatLxBI9gBamrtHrhAL1wy0L2yHvtyaeHbnfgDOvwBHrxAJfwnaebbnrfifHhDYfgasaacH8akY=wiFfYdH8Gipec8Eeeu0xXdbba9frFj0=OqFfea0dXdd9vqai=hGuQ8kuc9pgc9s8qqaq=dirpe0xb9q8qiLsFr0=vr0=vr0dc8meaabaqaciaacaGaaeqabaWaaeGaeaaakeaaimaacqWFaeFqaaa@3821@←WITH_DISTINCT_ENDS (*a*, A
 MathType@MTEF@5@5@+=feaafiart1ev1aaatCvAUfKttLearuWrP9MDH5MBPbIqV92AaeXatLxBI9gBamrtHrhAL1wy0L2yHvtyaeHbnfgDOvwBHrxAJfwnaebbnrfifHhDYfgasaacH8akY=wiFfYdH8Gipec8Eeeu0xXdbba9frFj0=OqFfea0dXdd9vqai=hGuQ8kuc9pgc9s8qqaq=dirpe0xb9q8qiLsFr0=vr0=vr0dc8meaabaqaciaacaGaaeqabaWaaeGaeaaakeaaimaacqWFaeFqaaa@3821@)

         **end if**

      **end if**

   **end for**

   **return ***A*

end function

The HSP_IS_CONTAINED routine returns true if the HSP provided as its first argument is contained in the HSP provided as its second argument. An HSP is considered to be contained in a second HSP if its query and subject bounds are contained in the query and subject bounds of the second HSP and if the second HSP has equal or higher score.

The WITH_DISTINCT_ENDS routine adds the alignment *a *to the set of alignments A
 MathType@MTEF@5@5@+=feaafiart1ev1aaatCvAUfKttLearuWrP9MDH5MBPbIqV92AaeXatLxBI9gBamrtHrhAL1wy0L2yHvtyaeHbnfgDOvwBHrxAJfwnaebbnrfifHhDYfgasaacH8akY=wiFfYdH8Gipec8Eeeu0xXdbba9frFj0=OqFfea0dXdd9vqai=hGuQ8kuc9pgc9s8qqaq=dirpe0xb9q8qiLsFr0=vr0=vr0dc8meaabaqaciaacaGaaeqabaWaaeGaeaaakeaaimaacqWFaeFqaaa@3821@ if and only if A
 MathType@MTEF@5@5@+=feaafiart1ev1aaatCvAUfKttLearuWrP9MDH5MBPbIqV92AaeXatLxBI9gBamrtHrhAL1wy0L2yHvtyaeHbnfgDOvwBHrxAJfwnaebbnrfifHhDYfgasaacH8akY=wiFfYdH8Gipec8Eeeu0xXdbba9frFj0=OqFfea0dXdd9vqai=hGuQ8kuc9pgc9s8qqaq=dirpe0xb9q8qiLsFr0=vr0=vr0dc8meaabaqaciaacaGaaeqabaWaaeGaeaaakeaaimaacqWFaeFqaaa@3821@ does not already contain an equal- or higher-scoring alignment that shares an endpoint with *a*. If *a *is added to A
 MathType@MTEF@5@5@+=feaafiart1ev1aaatCvAUfKttLearuWrP9MDH5MBPbIqV92AaeXatLxBI9gBamrtHrhAL1wy0L2yHvtyaeHbnfgDOvwBHrxAJfwnaebbnrfifHhDYfgasaacH8akY=wiFfYdH8Gipec8Eeeu0xXdbba9frFj0=OqFfea0dXdd9vqai=hGuQ8kuc9pgc9s8qqaq=dirpe0xb9q8qiLsFr0=vr0=vr0dc8meaabaqaciaacaGaaeqabaWaaeGaeaaakeaaimaacqWFaeFqaaa@3821@, then WITH_DISTINCT_ENDS filters A
 MathType@MTEF@5@5@+=feaafiart1ev1aaatCvAUfKttLearuWrP9MDH5MBPbIqV92AaeXatLxBI9gBamrtHrhAL1wy0L2yHvtyaeHbnfgDOvwBHrxAJfwnaebbnrfifHhDYfgasaacH8akY=wiFfYdH8Gipec8Eeeu0xXdbba9frFj0=OqFfea0dXdd9vqai=hGuQ8kuc9pgc9s8qqaq=dirpe0xb9q8qiLsFr0=vr0=vr0dc8meaabaqaciaacaGaaeqabaWaaeGaeaaakeaaimaacqWFaeFqaaa@3821@ to remove any lower-scoring alignments that share an endpoint with *a*. In this fashion, repeatedly calling the routine WITH_DISTINCT_ENDS ensures that the final list of alignments does not contain an alignment that shares an endpoint with a higher-scoring alignment. When two alignments share the same endpoint, the higher-scoring one is the preferred alignment; the lower-scoring alignment is a suboptimal artifact of the BLAST heuristics.

The x-drop algorithm requires a starting point (*p*_*q*_, *p*_*s*_) that will force an alignment between offset *p*_*q *_in the query and *p*_*s *_in the subject. It computes an alignment in both directions starting from this point. A starting point is defined for each HSP that is realigned. If possible, the starting point that was originally used to compute the HSP is reused. Due to the effects of SEG filtering and the newly computed scoring matrix, however, the previous starting point may no longer be desirable; it may lie in a region of nonpositive score. We discuss the rule used to validate the existing starting point, and if necessary choose a new one, in Additional file 3: tblastn_suppl.pdf.

Finally, we remark that Algorithm 1 is also correct pseudocode for BLASTP, which performs protein-query, protein-database searches. The difference is that for BLASTP there is only one window for each subject sequence: the window that includes the entire sequence. Moreover, for BLASTP the composition of the entire subject sequence is always used when performing compositional adjustment. Therefore, the compositionally adjusted matrix is necessarily the same for each HSP in a window and need only be computed once. In practice, the same code is used for both TBLASTN and BLASTP to implement Algorithm 1, but for BLASTP a conditional is used to ensure the matrix is only computed once for each window.

### Test sets and programs used

We describe below the specific executables, data sets, and methods used to generate the results presented in this paper. The variants of TBLASTN reported here were written in C, and, as noted below, some variants are available as part of the NCBI C and C++ software distributions; the computational modules involved are mirrored between the two distributions. Numerous auxiliary programs used to automate testing and summarize results were written in C, Perl, Python, and Bourne shell script.

### Executables used

TBLASTN is a mode of operation for the blastall executable. This executable is available for download from[36]. The C-TBLASTN and S-TBLASTN variants are available as a set of options to the blastall executable. S-TBLASTN is invoked using the command-line options "-p tblastn -F F -C 1". C-TBLASTN is invoked using similar options, but with "-C 1" replaced by "-C 2". B-TBLASTN is not currently available as a set of command line options. TBLASTN may be run without composition-based statistics, by omitting the "-C" option, but the default version runs with lower precision than B-TBLASTN. Executables that run B-TBLASTN and the specific versions of S-TBLASTN and C-TBLASTN used in this paper are available for download at[51].

The blastall executable by default uses BLOSUM62 to perform alignments of amino acid sequences, and this is the matrix used in all stages before composition adjustment is performed. The "-F F" option disables SEG filtering of the query sequence. SEG filtering of the subject sequence is on by default in any of the composition adjustment modes. We consider filtering both sequences to be unnecessary; when we tried filtering both sequences, we saw no improvement in statistical accuracy, but did see a decline in the ROC scores (data not shown).

### Tests using randomly permuted queries

To measure how effective composition-based statistics is at eliminating false matches with low E-value, we performed a series of tests using randomly permuted amino acid sequences from the mouse (*Mus musculus*) genome. One thousand protein sequences were randomly selected from the list of RefSeq[52] mouse proteins current on January 10, 2006. Sequences were permuted using their GenBank identification number as a seed to a random number generator. The permuted sequences are provided as Additional file 1.

We aligned the permuted sequences to a database of chromosomal sequences from the reference assembly of build 35 of the human (*Homo sapiens*) genome, released August 26, 2004. The database includes chromosomes X and Y and the unplaced sequence fragments included in the build. We omitted the mitochondrial genome from the database, however, as these sequences are known (see[42]) to have a different genetic code than nuclear DNA.

### ROC score tests on the yeast genome

To test the effectiveness of various modes of composition adjustment for TBLASTN, we performed a number of tests using the yeast nuclear genome. We downloaded the yeast genome from[53], a site containing reference genomes curated by NCBI staff. The version of the genome that we used was created on May 16, 2005.

We aligned a set of 102 protein domains to the yeast nucleotide genome using TBLASTN. This test set was first developed for the study in [11]. An updated version was used in [4], in which a human curated list of true positive matches to the yeast proteome was used to generate ROC scores. For the tests described here, we updated the true positive list to reflect changes in the published yeast genome. The updated list contains 987 query-subject matches to 894 distinct subject sequences. The version of the test set used in this paper is provided as Additional file 2.

In the yeast genome, each known yeast protein is annotated with the location and strand of its coding region. These annotations allow us to adapt the test set for use with TBLASTN as follows. For TBLASTN, alignments are divided into three categories: (1) alignments that match a query to the coding region of a known true positive match; (2) alignments that match a query to a known coding region that is not a true positive match; and (3) alignments that do not match a known coding region. An alignment is said to match a query to a coding region if the subject portion of the alignment overlaps the coding region and is on the same strand.

It is not uncommon for there to be more than one alignment between a query and a coding region. Indeed this is expected; protein-protein searches also report multiple alignments between pairs of proteins. When there is more than one alignment to a coding region, only the lowest E-value alignment between a particular query and the coding region is used when computing ROC scores. No attempt is made to apply a similar rule to noncoding regions. All alignments that do not overlap a coding region are categorized as false positive matches and counted when computing ROC scores.

We made two explicit exceptions to this scheme for classifying hits. The first exception is to add a particular pseudogene (Entrez Gene ID 850644) to our list of coding regions and to make the pseudogene a true positive for one of our queries, raising the maximum possible number of true positives to 988. Each of the variants tested found an alignment to this pseudogene with E-value smaller than 10^-12^. The pseudogene is expressed and produces a functional protein under certain conditions [54-56]. Though this region is labeled as a pseudogene, we do not believe an alignment algorithm should be expected to distinguish it from a true gene. The second exception is to categorize a particular alignment that overlaps one true positive coding region and one false positive coding region as a true positive match. This overlap is reported by all three variants of TBLASTN.

## Authors' contributions

EMG made most of the changes to the BLAST software, conducted most of the testing, and wrote the paper with assistance from SFA. Y-KY developed the method for C-BLAST and aided in the testing. RA provided resources and aided in the testing. AAS provided resources, made some of the changes to the BLAST software and developed earlier versions of the test sets. SFA directed the study and designed some of the methods. All authors edited, read, and approved the final manuscript.

## Supplementary Material

Additional File 1 Permuted mouse sequences. Sequence data used to test the statistical accuracy of TBLASTN. These data are not suitable for inclusion in a sequence database, as they have been randomly permuted and no longer represent true biological sequences. See the README included in the archive. Click here for file

Additional File 2 Yeast data set. queries and lists of true positives for performing ROC analysis of translated nucleotide search algorithms. Also contains the exact version of the yeast genome used in the experiments described in this paper. Click here for file

Additional File 3 Supplemental information. discusses rules for the treatment of nonstandard amino acids and for determining a staring point for gapped alignment when the current starting point is unacceptable. Click here for file
