# Supplementary material for: Composition-based statistics and translated nucleotide searches: Improving the TBLASTN module of BLAST
Source: BMC Biol. 2006 Dec 7;4:41. doi: 10.1186/1741-7007-4-41 (PMC1779365; doi:10.1186/1741-7007-4-41)
Supplement: Additional File 3 — Supplemental information. discusses rules for the treatment of nonstandard amino acids and for determining a staring point for gapped alignment when the current starting point is unacceptable. [file 1741-7007-4-41-S3.pdf]

Supplemental Information

Treatment of nonstandard characters

The amino acid alphabet used by BLAST has 28 characters, most of which may be represented in text by uppercase Roman letters. The alphabet contains characters that correspond to the 20 amino acids present in the known genetic codes. We refer to the remaining eight characters as nonstandard characters. The two nonstandard characters that occur often in TBLASTN searches are the stop character and the X character, and we discuss the treatment of these characters in Methods. The alphabet contains a nonstandard character that represents a gap in an alignment, but this character should not occur in any input sequence to TBLASTN. While none of the remaining five characters may occur in a translated sequence, some may occur in an amino acid sequence used as a query. Therefore, scores for alignments involving these characters must be defined.

Two of the nonstandard characters are true amino acids that, while not represented in any known genetic code, are inserted into certain proteins as part of translation of mRNA. These amino acids are selenocysteine [1, 2], represented by the character U, and pyrrolysine [3], represented by the character O. Selenocysteine and pyrrolysine residues are rare but are biologically important when they occur. Because the amino acids are so rare, it is difficult to assign aligned amino acid pairs involving these characters a meaningful score. BLAST chooses to, in effect, filter these characters from sequences; the score for aligning any character to either O or U is precisely the same as the score for aligning the character to X.

The remaining three characters represent ambiguity between pairs of amino acids: B represents an ambiguity between aspartic acid and asparagine; J represents an ambiguity between leucine and isoleucine; and Z represents an ambiguity between glutamic acid and glutamine. These characters may occur in the query amino acid sequence if the method used to obtain the sequence could not distinguish between two amino acids. For instance, mass spectrometry cannot distinguish leucine from isoleucine.

S-TBLASTN simply scales the scores for alignments involving two-letter ambiguity characters in exactly the same fashion as it scales the scores of the standard amino acids. C-TBLASTN computes scores for B, J, and Z using the target frequencies and background probabilities computed while optimizing the scores of the standard amino acids. Similar formulas are used for each of B, J, and Z; we only describe the formulas used when calculating scores involving B here.

Let Q_{ij}, P_i, and P'_i be calculated as in [4] for all standard amino acids i and j. Q_{ij} represents the target frequency of substituting amino acid i in the query with amino acid j in the subject. P_i and P'_i represent the background frequencies of amino acid i in the query and subject sequences, respectively. Let λ be a statistical parameter that gives the scale of the scoring system. The score of aligning B in the query to standard amino acid j in the subject is

$$\text{score(B, } j) = \frac{1}{\lambda} \ln \frac{Q_{Dj} + Q_{Nj}}{(P_D + P_N)P'_j}, \tag{S1}$$

where D represents aspartic acid, and N represents asparagine. The score for aligning B in the query with X is computed using the formula

$$\begin{aligned} &\text{score(B, X)} && \text{(S2)} \\ &= \min \left\{ -1, \textstyle\sum_{j \in \mathcal{S}} \text{score}(B, j) \times P'_j \right\}. \end{aligned}$$

If j is one of the characters B, J, or Z, representing an ambiguity between characters k and ℓ, then

$$\begin{aligned} &\text{score(B, } i) \\ &= \frac{1}{\lambda} \ln \frac{Q_{Dk} + Q_{D\ell} + Q_{Nk} + Q_{N\ell}}{(P_D + P_N)(P'_k + P'_\ell)}. \end{aligned} \tag{S3}$$

BLAST uses integer scores, so the results of equations (S1)-(S3) are rounded the nearest integer before they are used. Occurrences of B in the subject sequence are treated analogously.

We remark that the characters J and O are recent additions to the alphabet used by BLAST and may not be supported in older versions of BLAST or in all modes of operation.

Determining a starting point for a gapped alignment

All variants of TBLASTN discussed in this paper apply SEG filtering to the translated subject sequence before applying compositional adjustment. Earlier stages of the BLAST algorithm, and in particular the stage that generates starting points for gapped alignments, do not filter the subject sequence. Once the subject sequence has been filtered, a starting point may no longer be desirable because it lies in a region

that has been overwritten with Xs. Furthermore, compositional adjustment itself can effect the quality of a given starting point. Therefore, TBLASTN must test each existing starting point to determine whether it is an acceptable location to start a gapped alignment, and if not must compute a new starting point.

To determine whether a starting point from previous stages of the BLAST algorithm is acceptable, TBLASTN uses the compositionally adjusted scoring system to calculate the score of an ungapped alignment that contains the starting point. Usually, the ungapped alignment extends five positions to the left and five positions to the right of the starting point, but it may be shorter if there is not enough sequence data to extend that far. If the score of this ungapped alignment is positive, then the starting point is acceptable and is used to recompute a gapped alignment.

If the starting point is not acceptable, then TBLASTN computes a new starting point using the algorithm outlined in the following pseudocode. In the pseudocode, q and s represent the query and subject data, respectively. The interval $[\ell_q, r_q]$ is the range of a particular HSP in the query, and $[\ell_s, r_s]$ is its range in the subject. M is a compositionally adjusted scoring matrix and UNGAPPED_SCORE(M, q, s, i, j, n) is a function that computes the score of an ungapped alignment of length n starting at $(q[i], s[j])$.

Algorithm 2. Find a starting point for gapped alignment.

function FIND_GAPPED_START(M, q, ℓ_q, r_q, s, ℓ_s, r_s)
    max_score ← 0; max_index ← 0
    length ← $\min\{r_q - \ell_q + 1,\ r_s - \ell_s + 1\}$
    for $i \leftarrow 0$ **to** length − 11 **do**
        S ← UNGAPPED_SCORE(M, q, s, $i + \ell_q$, $i + \ell_s$, 11)
        if $S >$ max_score **then**
            max_score ← S
            max_index ← $i + 5$
        end if
    end for
    return (max_index + ℓ_q, max_index + ℓ_s)
end function

Algorithm 2 computes the score of several ungapped alignments of length 11, and if any of these has positive score, then it chooses the midpoint of the highest scoring alignment to be a new starting point for gapped alignment. It can, and in practice does, happen that none of the alignments tested has positive score. In this case, the left endpoint of the HSP itself is used as the starting point for gapped alignment.

References

1. Zinoni F, Birkmann A, Leinfelder W, Böck A: **Cotranslational insertion of selenocysteine into formate dehydrogenase from *Escherichia coli* directed by a UGA codon**. *Proc. Natl. Acad. Sci. USA* 1987, **84**:3156–3160.
2. Low SC, Berry MJ: **Knowing when not to stop: Selenocysteine incorporation in eukaryotes**. *Trends Biochem. Sci.* 1996, **21**:203–208.
3. Hao B, Gong W, Ferguson TK, James CM, Krzycki JA, Chan MK: **A New UAG-Encoded Residue in the Structure of a Methanogen Methyltransferase**. *Science* 2002, **296**:1462–1466.
4. Altschul SF, Wootton JC, Gertz EM, Agarwala R, Morgulis A, Schäffer AA, Yu YK: **Protein database searches using compositionally adjusted substitution matrices**. *FEBS J.* 2005, **272**:5101–5109.
